# Renal morphology and vascularization in Margay (*Leopardus wiedii)* (Carnivora: Felidae): report of 02 cases

**DOI:** 10.29374/2527-2179.bjvm003425

**Published:** 2025-08-11

**Authors:** Anieli Vidal Stocco, Shirley Viana Peçanha, Marcelo Salvador Gomes, Carlos Augusto Santos Sousa, Paulo Souza-Júnior, Marcelo Abidu-Figueiredo

**Affiliations:** 1 Programa de Pós-graduação em Medicina Veterinária, Departamento de Medicina e Cirurgia Veterinária, Instituto de Veterinária, Universidade Federal Rural do Rio de Janeiro. Seropédica, RJ, Brazil.; 2 Programa de Pós-graduação em Medicina Veterinária, Universidade Federal Fluminense. Niterói RJ. Brazil.; 3 Universidade Augusto Mota (UNISUAM), Rio de Janeiro, RJ, Brazil.; 4 Instituto de Ciências Agrárias, Universidade Federal dos Vales do Jequitinhonha e Mucuri (UFVJM), Unaí, MG, Brazil.; 5 Departamento de Medicina Veterinária, Universidade Federal do Pampa (UNIPAMPA), Uruguaiana, RS, Brazil.; 6 Departamento de Anatomia Animal e Humana, Universidade Federal Rural do Rio de Janeiro, Seropédica, RJ, Brazil.

**Keywords:** anatomy, wild cats, urinary system, anatomia, felinos silvestres, sistema urinário

## Abstract

The margay (*Leopardus wiedii*) is a neotropical, arboreal wildcat widely distributed in Brazil. Since 2014, it has been classified as Vulnerable on the National List of Endangered Species (ICMBio) and listed as Near Threatened by the IUCN. Morphological studies of wild felids are essential for understanding their ecological and functional adaptations to their environment. Anatomy is a fundamental tool for investigating aspects related to species biology and evolution, particularly interactions with the environment, diet, and reproduction. This report describes the renal morphology and vascularization of *Leopardus wiedii*, with a focus on potential clinical and surgical applications, including vascular anastomoses, imaging studies, nephrectomies, and preoperative planning to minimize complications. The animals were formalin-fixed at the Laboratory of Teaching and Research in Morphology of Domestic and Wild Animals at the Federal Rural University of Rio de Janeiro, injected with colored latex, and dissected. The right kidney measured 4.68 × 2.55 × 2.34 cm and the left kidney measured 4.47 × 2.73 × 2.59 cm in animal 01, and the right kidney measured 3.32 × 2.10 × 2.34 cm and the left kidney measured 3.69 × 2.26 × 2.20 cm in animal 02. Single renal arteries were observed bilaterally, with a single renal vein on the left and double veins on the right. These findings enhance the anatomical knowledge of the species and support its clinical and conservation management.

## Resumo

O gato-maracajá (*Leopardus wiedii*) é um felino silvestre arbóreo neotropical, amplamente distribuído no Brasil. Desde 2014, é consideradoVulnerável pela Lista Nacional de Espécies Ameaçadas de Extinção (ICMBio) e classificado como Quase Ameaçado pela IUCN. Estudos morfológicos em felinos silvestres são fundamentais para entender suas adaptações ecológicas e funcionais. A anatomia é uma ferramenta essencial para investigar aspectos relacionados à biologia e evolução das espécies, especialmente no que se refere às interações com o ambiente, dieta e reprodução. Este relato descreve a morfologia e vascularização renal de *Leopardus wiedii*, com foco em possíveis aplicações clínico-cirúrgicas, como anastomoses vasculares, exames de imagem, nefrectomias e planejamento pré-operatório visando reduzir complicações. Os animais foram formolizados no Laboratório de Ensino e Pesquisa em Morfologia dos Animais Domésticos e Selvagens da Universidade Federal Rural do Rio de Janeiro, injetados com látex colorido e dissecados. Os rins mediram: 4,68 x 2,55 x 2,34 cm (direito) e 4,47 x 2,73 x 2,59 cm (esquerdo) no animal 01; 3,32 x 2,10 x 2,34 cm (direito) e 3,69 x 2,26 x 2,20 cm (esquerdo) no animal 02. Observaram-se artérias renais únicas bilateralmente, veias únicas nos rins esquerdos e duplas nos direitos. Esses achados ampliam o conhecimento anatômico da espécie e contribuem para seu manejo clínico e conservacionista.

## Introduction

South American felids comprise 10 species that have adapted to various habitats, ranging from the Andean Mountains to the flooded savannas of the Pantanal. Based on an average body mass of less than 20 kg, eight of these species are classified as “small cats,” including the ocelot (*Leopardus pardalis*), margay (*L. wiedii*), and oncilla (*L. tigrinus)* (Nowak, 1999). Despite their genetic, morphological, and behavioral differences, all South American felids face challenges that threaten their survival. Animals in the wild continue to be hunted illegally, particularly for their fur and the pet trade. Additionally, pervasive habitat loss is fragmenting and degrading their home ranges. The biology and status of most wild cats, especially smaller species, remain unknown. The margay is categorized as “near threatened” and is experiencing a global decline (Oliveira et al., 2015).

Morphological studies of wild felines are important because they contribute to our understanding of the ecological characteristics of a species. Anatomy is a crucial tool for answering questions about species biology and evolution, especially those concerning interactions with the habitat, environment, diet, and reproductive adaptations.

Each kidney has a cranial and caudal pole, medial and lateral borders, and dorsal and ventral surfaces. The convex lateral border connects the cranial and caudal poles. The medial border has an indentation (hilum) that defines the renal sinus. The renal sinus contains the ureter, renal artery, renal vein, lymphatic vessels, and nerves. Of these structures, the renal artery is the most dorsal, and the renal vein is the most ventral (Evans & De Lahunta, 2013). In most species, the right renal artery is typically more cranial than the left because of the relatively more cranial position of the right kidney (Nickel & Schummer, 1981).

Knowledge of the reference values for kidney measurements can aid in the diagnosis of various renal diseases. Alterations in these measurements may indicate nephropathies due to hypertrophic processes and/or atrophy (Beland et al., 2010; Yamashita et al., 2015). Therefore, establishing a standard for typical renal measurements in each species is imperative. Necropsy studies in humans suggest that variations in kidney size and weight are sex-related, with larger kidneys observed in males. Regardless of sex, the left kidney is larger than the right kidney (Moell, 1956). However, this information is unclear for wild animals. Jarreta et al. (2004) evaluated the kidneys of small-spotted cats using ultrasonography and found no differences in measurements between antimers or sexes. Similar findings were reported in the serval (*Leptailurus serval*) by Hespel et al. (2019).

Despite the increase in basic and applied research involving wild animals, anatomical studies describing kidney anatomy and vascularization in these species remain scarce. Regarding morphology, however, a limited number of studies on these species have demonstrated the importance of obtaining anatomical information for future research and species preservation in human care or natural habitats. Thus, this study determined the morphometric variables of the kidneys and renal vessels in the margay (*Leopardus wiedii*) and compared the data with an emphasis on the order Carnivora.

## Material and methods

During dissection activities at the Laboratory of Teaching and Research in Morphology of Domestic and Wild Animals (LEPeMADS) in the Department of Animal and Human Anatomy at the Federal Rural University of Rio de Janeiro, renal morphology and vascularization were observed in two adult male margay specimens. The specimens were donated by Serra dos Órgãos National Park.

The cadavers were identified and positioned in the right lateral decubitus. Subsequently, the thorax was opened and dissected to expose the thoracic aorta, into which a number 6 urethral catheter was inserted. The arterial system was then flushed with 0.9% saline solution and fixed with 10% formalin solution. Next, an aqueous solution (1:1 dilution) of Petrolátex S-65 (Refinaria Duque de Caxias—REDUC, Petrobrás, Duque de Caxias-RJ), mixed with a dye (Suvinil Xadrez®), was injected through the catheter. The cadavers were then immersed in a 50-liter low-density polyethylene container filled with 10% formalin solution to complete the latex fixation and polymerization process. Seven days after the latex injection, the specimens were washed with running water. The peritoneal cavity was opened and dissected to expose the kidneys. A digital caliper (Eda brand) was used to obtain renal measurements, including length, width, thickness, and ellipsoid volume (Sampaio, 1995), and the lengths of the renal veins and arteries.

## Description of the cases

### Animal 01

The young adult male animal had a rostral-sacral length of 51 cm. The right kidney measured 4.68 cm in length, 2.55 cm in width, and 2.34 cm in thickness. It had an ellipsoid volume of 14.60 cm^3^ and was located between the second and fifth lumbar vertebrae (L2-L5). Two renal veins were observed in this kidney: one craniodorsal and one caudoventral, both at the L4 level. The craniodorsal renal vein near the renal hilum was formed by the confluence of two interlobar veins: a dorsal vein measuring 0.94 cm in length and a ventral vein measuring 0.62 cm in length, which merged into a renal vein measuring 0.95 cm. The second, caudoventral renal vein, measured 0.98 cm in length. The renal artery was single and measured 3.07 cm at the L3-L4 level and gave off branches to the ureter and right adrenal gland.

The left kidney measured 4.47 cm in length, 2.73 cm in width, and 2.59 cm in thickness. It had an ellipsoid volume of 16.52 cm^3^ and was located between L2 and L5. The left kidney had a single renal vein formed by the confluence of two interlobar veins: a dorsal vein measuring 1.71 cm in length and a ventral vein measuring 1.84 cm. These veins merged into a renal vein measuring 1.35 cm at the L4 level, which received drainage from the left testicular vein. The single renal artery measured 2.53 cm in length (L4) and gave off branches to the ureter and left adrenal gland. It was divided into two branches for the renal hilum: a dorsal branch measuring 0.81 cm and a ventral branch measuring 0.80 cm.

**Figure 1:**
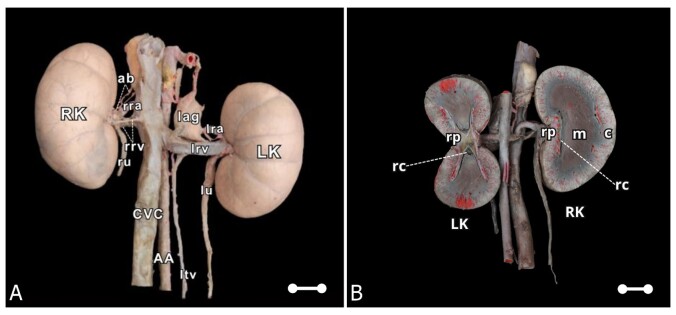
(A) Digital photomacrograph showing the ventral view of the kidneys and their respective renal vessels in the margay (Animal 01). CVC: caudal vena cava; AA: abdominal aorta; RK: right kidney; LK: left kidney; lag: left adrenal gland; rrv: double right renal vein; lrv: left renal vein; rra: right renal artery; lra: left renal artery; ltv: left testicular vein; ru: right ureter; lu: left ureter; ab: adrenal branches; (B) Digital photomacrograph of the transverse and longitudinal sections of the margay kidney (Animal 02). RK: right kidney; LK: left kidney; c: renal cortex; m: renal medulla; rc: renal crest; rp: renal pelvis. Scale bar: 1 cm.

The kidneys had cranial and caudal extremities, and lateral and medial margins (renal hilum). They also had dorsal and ventral surfaces. Their surfaces were smooth, devoid of lobulations, and exhibited shape symmetry but positional asymmetry. The kidneys consisted of an outer renal cortex and an inner medulla. The renal pelvis and crest were visible in the longitudinal and transverse sections. The kidneys were simple and unipapillary (Figure 1A).

### Animal 02

The second male young adult animal measured 53.3 cm in rostral-sacral length. The right kidney measured 3.32 cm in length, 2.10 cm in width, and 2.34 cm in thickness. It had an ellipsoid volume of 8.53 cm^3^ and was located between the second and fourth lumbar vertebrae (L2–L4). Two renal veins were observed at the L3 level: one craniodorsal and the other caudoventral. The caudoventral vein was formed by the confluence of two interlobar veins: a dorsal vein measuring 0.66 cm and a ventral vein measuring 0.78 cm. These veins merged into a renal vein measuring 0.84 cm. The second, craniodorsal renal vein, was 1.22 cm long. The renal artery was single and measured 2.51 cm at the L3 level and gave off branches to the ureter and right adrenal gland. It was then divided into two branches for the renal hilum: a cranial branch measuring 0.57 cm and a caudal branch measuring 0.51 cm.

The left kidney measured 3.69 cm in length, 2.26 cm in width, and 2.20 cm in thickness. It had an ellipsoid volume of 9.59 cm^3^ and was located between L2 and L4. It had a single renal vein (L2) that measured 2.24 cm in length and received drainage from the left testicular vein. The renal artery was single and measured 1.27 cm in length at the L3 level. It gave off branches to the ureter, adrenal gland, and lumbar musculature and was divided into two branches for the renal hilum: a cranial branch measuring 0.85 cm and a caudal branch measuring 0.66 cm.

The kidneys had cranial and caudal extremities, as well as lateral and medial margins (renal hilum). They also had dorsal and ventral surfaces. Their surfaces were smooth, devoid of lobulations, and exhibited shape symmetry but positional asymmetry. The kidneys consisted of an outer renal cortex and an inner medulla, with the renal pelvis and crest visible in the longitudinal and transverse sections (Figure 1B). They were simple and unipapillary (Figure 1A).

## Discussion

The kidneys of the margay had capsular veins, were smooth, and lacked lobation, similar to the findings observed in the domestic cat (Stocco et al., 2016), ring-tailed coati (Duarte et al., 2022), crab-eating fox (Souza-Junior et al., 2020), pampas fox (Souza et al., 2018), puma (Stocco et al., 2025), leopard (Chandrasekhar et al., 2015), and clouded leopard (Doley et al., 2015).

In transverse and longitudinal sections, the kidneys presented two distinct regions: a peripheral cortical region and a central medullary region. They were also unipyramidal (unipapillary), in accordance with the descriptions by Stocco et al. (2016) (domestic cat), Duarte et al. (2022) (ring-tailed coati), Souza-Junior et al. (2020) (crab-eating fox), Souza et al. (2018) (pampas fox), Chandrasekhar et al. (2015) (leopard), Doley et al. (2015) (clouded leopard), Wilson et al. (2024) (tiger), and Stocco et al. (2025) (puma).

The right kidney of the margay measured 4.68 × 2.55 × 2.34 cm, and the left kidney measured 4.47 × 2.73 × 2.59 cm in animal 01. The right kidney measured 3.32 × 2.1 × 2.34 cm, and the left kidney measured 3.69 × 2.26 × 2.20 cm in animal 02. These are smaller than the kidneys of domestic dogs, which measure approximately 60–90 × 40–50 × 30–40 mm (Evans & De Lahunta, 2013), and leopards, whose right and left kidneys measure 7.11 × 5.35 × 3.57 cm and 7.14 × 4.88 × 3.37 cm, respectively (Sarma et al., 2004). They are also larger than the kidneys of other carnivores, such as *Nasua nasua* 30 × 16 × 13 mm (Duarte et al., 2022), and *Mustela putorius furo* 24–30 × 12–13.5 × 11–13.5 mm (Evans & An, 2014). The values obtained from the margay are similar to those from other carnivores, such as *Lycalopex gymnocercus* (45 × 24 × 21 mm; Souza et al., 2018), *Cerdocyon thous* (43-55 × 21-29 × 18-30 mm; Souza Junior et al., 2020), and domestic cats (38 × 24 × 23 mm; Stocco et al., 2016). These differences in renal measurements among carnivores may be correlated with body size (Duarte et al., 2022).

Huaijantug et al. (2017) conducted a renal ultrasound study of tigers and found that the obtained measurements showed a statistically significant difference in body weight and renal length between genders. The right kidney was significantly longer than the left kidney (10.23 ± 0.76 cm in males vs. 9.94 ± 0.80 cm in females; *P* < 0.05). The study suggests that kidney length is significantly associated with body weight and has a positive linear relationship with it.

Renal measurements are important for making clinical decisions and serve as indicators of the renal functional reserve (Moorthy & Venugopal, 2011). Morphometric values also provide indirect insights into the progression and stability of kidney disease. On renal ultrasonography, end-stage chronic kidney disease in cats is characterized by irregular kidney contours and reduced dimensions (Griffin, 2020). Chronic renal failure is becoming increasingly common in wild felines, particularly those under human care (D’Arcy, 2018; Mitchell et al., 2018). However, information regarding renal dimensions in species commonly treated in wildlife medicine is scarce.

Among the renal diseases found in nature, *Dioctophyma renale* parasitism has been reported in *Leopardus pardalis* (Goossen et al., 2022) and *Leopardus geoffroyi* (Trindade et al., 2018). This nematode predominantly affects the right kidney, leading to renal parenchyma destruction (Measures, 2001; Pedrassani & Nascimento, 2015). *Dioctophyma renale* infection can be diagnosed using ultrasonography because it causes changes in renal dimensions and loss of distinction between the cortex and medulla (Mesquita et al., 2014). Recently, Wilson et al. (2024) described a case of renal candidiasis in a captive tiger. These findings may influence renal measurements and are important factors to consider in morphological and functional evaluations.

The renal measurements presented in this study can serve as preliminary parameters for interpreting the imaging and necropsy findings in margays. However, these data should be applied with caution, as the body size of a particular specimen may result in normal kidneys that are larger or smaller than those proposed in our study. For example, the kidneys from the southern subpopulation of *C. thous* were significantly larger than those from the Southeast, likely due to differences in body size and diet (Souza-Junior et al., 2020).

Regarding renal artery irrigation, both kidneys presented a single artery that emerged directly from the abdominal aorta. This finding is consistent with the results obtained by Stocco et al. (2016) (domestic cat), Duarte et al. (2022) (ring-tailed coati), Souza-Junior et al. (2020) (crab-eating fox), and Souza et al. (2018) (pampas fox). However, duplication of the renal artery has been observed in domestic cats (Pestana et al., 2011) and crab-eating foxes (Viana-Peçanha et al., 2020).

Regarding venous drainage, the renal veins empty directly into the caudal vena cava, which is consistent with findings in other carnivores: Stocco et al. (2016) in domestic cats, Souza et al. (2018) in pampas foxes, Souza Junior et al. (2020) in crab-eating foxes, Castano et al. (2022), Duarte et al. (2022) in ring-tailed coatis, and Stocco et al. (2025) in pumas. For kidney transplantation, the left kidney is preferred because of its longer renal vein, which facilitates anastomosis (Gregory et al., 1992; Gregory & Bernsteen, 2003; Budgeon et al., 2017).

In the margay, the left renal vein was single in both dissected animals; however, the right kidney showed renal vein duplication in both animals.

Variations in renal vein number have been reported in domestic cats (Campos et al., 2014; Stocco et al., 2014), ocelots (*Leopardus pardalis*) (Stocco et al., 2017), small wild cats (*Leopardus guttulus*) (Stocco et al., 2018), ring-tailed coatis (Duarte et al., 2019), and pumas (Castano et al., 2022; Stocco et al., 2025). These vascular variations have clinical significance, particularly in procedures such as nephrectomy. Failure to properly identify and ligate these vessels before transection poses a high risk of severe hemorrhage (Tillson & Tobias, 2018).

Further studies are needed, particularly with regard to sample size, to more completely characterize the renal anatomy of margays. Knowledge of precise renal anatomy and vascularization is important for radiology, ultrasonography, and surgery, and provides valuable information for the clinical treatment of this species, especially for professionals working in zoos and conservation units.

## Conclusion

The kidneys of the margay (*Leopardus wiedii*) have a smooth, non-lobulated external surface, which is consistent with renal morphology observed in other carnivores. Internally, a clear distinction between the cortical and medullary regions was evident, with an unipyramidal organization. The renal dimensions were within the expected range for various species, reflecting size variations that were likely associated with overall body mass. Regarding vascularization, the presence of a single renal artery and venous drainage pattern aligned with typical findings in other carnivores, although individual variations may occur. These anatomical characteristics improve our understanding of *Leopardus wiedii* and provide valuable insights into clinical and surgical applications. However, additional studies with larger sample sizes are necessary to establish a more detailed and representative anatomical profile of the renal structures in this species.
